# Trend Change Analysis as a New Tool to Complement the Evaluation of Human Body Balance in the Time and Frequency Domains

**DOI:** 10.5114/jhk/163058

**Published:** 2023-07-15

**Authors:** Piotr Wodarski

**Affiliations:** 1 Department of Biomechatronics, Faculty of Biomedical Engineering, Silesian University of Technology, Poland.

**Keywords:** index, postural stabilization, stabilography, TCI, MACD

## Abstract

Trend change analysis is a tool that complements the assessment of human body stability and provides information on the number and frequency of postural corrections during an examination. The present research aims to determine the possibility of using this method of analysis to investigate postural stability during tests of standing with open eyes (OE) and closed eyes (CE). A total of 118 participants (67 females, 51males) aged 23 (SD 1.3) were assessed. Tests involved standing on a stabilographic platform for 50 s. Trend change analysis was used to evaluate displacement values of the center of pressure (COP). Values for the COP courses as well as values associated with trend change analysis, such as: TCI, MACD_t and MACD_V were determined. Histograms of distribution were plotted for TCI values. The present study provides information on alterations of the strategy used for maintaining balance, which are associated with the number of postural corrections and COP displacement between corrections for measurements taken during the standing test with CE in relation to OE measurements. The strategy demonstrated an ability to detect a smaller number of quick corrections, an increased number of corrections of longer duration, and the elongation of displacement between subsequent postural corrections. Slight standard deviations in TCI and MACD_t values calculated during trend change analysis, for both the OE and CE conditions, made it possible to classify these values as indexes of postural stability with significant sensitivity to slight changes.

## Introduction

Humans have numerous regulatory mechanisms responsible for keeping a vertical body posture, and irregularities in the functioning of these mechanisms often lead to locomotor disorders that can result in falls and injuries (Błaszczyk et al., 2016; Gorwa et al., 2021; Hof et al., 2005). Understanding the causes of postural disorders and achieving proper diagnosis is therefore essential. Indeed, adequate and objective methodology for obtaining stabilographic measurement values that describe the functioning of regulatory systems involved in maintaining vertical body posture can provide information about the health condition of individuals being examined (Bibrowicz et al., 2018; Kamieniarz et al., 2021). In addition, periodic measurement of stabilographic values may be a useful tool for the objective evaluation of balance training (Bibrowicz et al., 2018; Kamieniarz et al., 2021; Wodarski et al., 2022).

Evaluation of stabilographic values has been carried out for several years and includes the assessment of the center of pressure (COP) displacement of the feet when placed on the ground, along with the center of mass (COM) displacement (Brachman et al., 2017; Bugnariu and Fung, 2007; Wodarski et al., 2022). Subsequent positions of the COP or COM recorded over time can then be analyzed as a whole or individually for the anterior-posterior (AP) and mediolateral (ML) directions (Paillard et al., 2006; Tan et al., 2015). From such assessments, a thorough analysis of the ability of individuals to maintain balance can then be conducted. Among the most frequently analyzed time measures are length of the support path (Path), COP movement velocity, the surface area of the ellipse of the load (Area), load symmetry, and range of COP motion (Range of Motion) (Błaszczyk, 2008; Jurkojć, 2018; Takakusaki, 2017). In addition to these measurements, entropy and frequency values can be calculated to supplement the data (Błażkiewicz et al., 2021; Chou et al., 2021). The latter method finds application in the investigation of factors disturbing the balance of cyclic characters, both in the form of a movable ground, and the oscillation of virtual surroundings in a virtual reality environment. In this way, a cyclic component of the COP or COM movement is observed. Its frequency is equivalent to the frequency of the balance-upsetting factor (Wodarski et al., 2020, 2022).

The most popular analyses in the scope of frequency include a fast Fourier transform (FFT), short-time Fourier transformation (STFT), and wavelet analysis (Nema et al., 2017; Wodarski et al., 2020). Due to the transformation of the COP signal into the frequency domain, it is possible to calculate the distribution of the signal components of given frequencies. These can then be compared with, for instance, a set model of visual disturbances, as was demonstrated in studies by Jurkojć (2018) and Cleworth et al. (2016). Previous research (Bizid et al., 2009; Micarelli et al., 2019; Wodarski et al., 2020, 2022) provided information on the method used for the interpretation of signals in the frequency domain. It has been proven that frequency components are related to the strategy of maintaining balance, where low frequencies (0–0.5 Hz) mostly account for visuo-vestibular regulation, medium frequencies (0.5–2 Hz) for cerebellar regulation and high frequencies (>2 Hz) for proprioceptive regulation (Bizid et al., 2009; Micarelli et al., 2019). As far as the frequency domain is concerned, force and force density can also be determined for certain frequency bands much easier than for time values, which require computations using band filtration (Jurkojć, 2018; Wodarski et al., 2022). Also, the distribution of force in the frequency domain provides information on which frequency the energy of COP movement is most concentrated. In addition, the application of wavelet analysis to the assessment of stability makes it possible to define force and amplitude for bands of very low frequencies (lower than 0.5 Hz), which gives this method an advantage over FFT and STFT. On the other hand, a drawback of wavelet analysis in relation to FFT and STFT is that there is an uneven distribution of frequency bands (Wodarski et al., 2020).

Without a doubt, frequency analyses provide additional information on the human motor system in comparison with traditional analytical methods in the time domain. However, it should be noted that they are based on the search for periodic components of COP displacement signals, which often have a sinusoidal shape, as is the case of FFT, STFT, and Coiflet wavelets (Wodarski et al., 2020).

A slightly different approach to the search for patterns of change in the COP signal is the analysis of structural values which are determined by the distribution of the COP signal into individual constituents. Such analyses also encompass sway density, which enables an indirect evaluation through the analysis of time, in which the COP remains in one location. This takes into account momentary changes in COP motion velocity values, but does not consider its directivity (Marco-Ahulló et al., 2022). Directional analysis may assess changes in velocity directions, or COP movement directions, separately in the AP and ML axes. The question then arises over how to extract only significant changes of direction, and how to determine which irregularities are non-significant momentary ‘jumps’ of the COP, and can therefore be filtrated. Such errors are connected with the methods of determining the COP location or result from computational approximations. An alternative solution can be provided through the analysis of trend changes in COP directional movement. Trend change analysis is an innovative approach that uses statistical methods drawn from the so-called ‘technical analysis’ of changes in the values of stocks and shares at the stock exchange (Wodarski et al., 2022). The methodology which was developed for the purposes of prediction of the behaviour of stocks and currency rates is also suitable for the prediction of values of COP movement signals as well as the detection of changes in the signal trend during an examination. Computations of adequate indexes were designed to omit local extremes of the signal that do not cause any change to its trend. The information obtained using these types of indexes and analysis is associated with the number of postural corrections during the examination. This method was previously assessed in dynamic tests that investigated the application of this innovative analysis to stabilographic tests using virtual reality (Wodarski et al., 2022).

**Figure 1:**
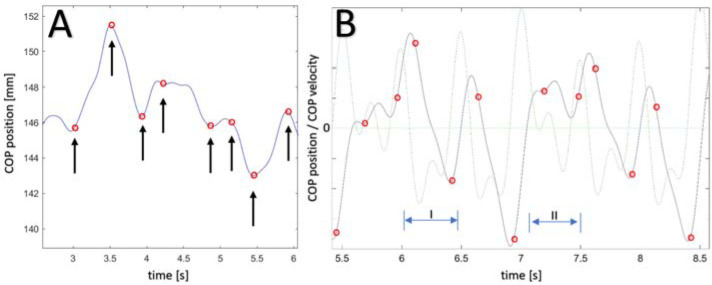
A) COP displacement signal with marked spots indicating trend changes, which were calculated by the MACD algorithm; B) COP displacement signal with marked spots indicating trend changes (in blue) and with the plotted course of COP momentary velocity changes (in green).

The trend change in the COP displacement in either the AP or ML directions can be defined as a significant change in the direction of COP movement, i.e., the change which does not have a short-term character that is non-significant to the conducted analysis (Figure 1A). In compliance with data available in the subject literature, non-significant changes are defined as changes with a frequency no higher than 5 Hz. This means that the time between subsequent trend changes will be shorter than 0.1 s, i.e., the time between trend changes may be considered the half of the period (Marco-Ahulló et al., 2022; Nema et al., 2017). The process for omitting non-significant constituents from the trend change detection algorithm is shown in Figure 1B, which compares changes in time and velocity values of COP coordinates.

Analysis of the data presented in Figure 1B demonstrates that time moments in which the trend change is discovered occur shortly after the change of COP displacement direction. Special attention should be paid to the fact that moments of trend change are not detected at moments of short-term changes of velocity, or moments of local extremes of the COP displacement signal. Time window I of Figure 1B, between 6 s and 6.5 s of movement, serves as an example of this. In this time bracket, a momentary change of the motion velocity vector occurred which did not disturb the operation of the trend change detection algorithm. Moreover, in time window II (Figure 1B) which spans 7.1–7.5 s, the trend change was detected slightly earlier than the change took place, which was caused by an earlier change in velocity and its constant rise through negative values. Therefore, the algorithm was designed to skip momentary oscillations of body balance (local extremes in the courses) that did not change the COP movement trend.

The present research aimed to investigate the possibility of using trend change analysis and the author’s Trend Change Index (TCI) for the examination of postural stability during free standing tests with open eyes (OE) and closed eyes (CE). In this regard, it was assumed that trend change analysis could also be applied to the evaluation of change to the strategy used for maintaining balance during CE testing. The OE and CE tests constitute a certain standard in the assessment of balance and are an essential part of the methodology used in diagnostics and in the prediction of falls (Mańko et al., 2016; Quijoux et al., 2021). The technical analysis of the COP signal based on the methods used in stock exchange analyses makes it possible to determine the number of postural corrections in the AP and ML directions as well as to define an average period of time between subsequent corrections of posture, which was indicated in previous research (Wodarski et al., 2022). The number of trend changes in the COP signal in time may translate into the number of reactions of the human body with postural disorders, which are indispensable for maintaining a vertical position during free standing (Sozzi et al., 2021; Takakusaki, 2017).

## Methods

### Participants

The study group included 118 participants (67 females and 51 males) aged 23 ± 1.3 years and with mean body mass of 71 ± 52 kg. None of the participants had a history of extreme lower limb injury or suffered from motor system dysfunctions or balance disorders.

This study was approved by the Ethics in Research Committee of the Academy of Physical Education in Katowice (number 5/2020).

### Measures

The measurement stand (FDM-S, Zebris Medical GmbH, Germany) consisted of a platform that enabled measurements of the distribution of pressure exerted by feet on the ground. The stand used a sampling frequency of 100 Hz, and 2560 tension meter sensors, with a sensor area of 34 cm x 54 cm.

### Design and Procedures

The testing procedure consisted of two measurements of free-standing on the platform, one of which was recorded with OE, and another with CE. Tests were conducted in random order. Participants had been informed about the procedures before testing. The test lasted 50 s, out of which a section of 30 s (starting at the 10^th^ s of the test) was subjected to analysis.

Methodology of the change trend determination is based on the computations applied to the technical analysis of the courses of stock rates at stock exchanges. To be more precise, it is an algorithm that enables the calculation of the MACD (Moving Average Convergence / Divergence) index, which is a popular tool among investors. The computations make use of the mechanism of a moving average (or rolling average) to detect moments in time in which a trend change of the tested signal occurs. The full algorithm used for such computations was developed and presented in Wodarski et al. (2022). As a result of the algorithm operation, information is obtained in the scope of moments of trend changes of the signal of the COP displacements in a selected direction (Figure 1A).

For the selected points in Figure 1A, the following values were calculated using the MACD computation methodology:

- TCI – A value for the total number of trend changes in the COP course during a measurement;
- MACD_t – time value between subsequently detected points of a trend change in the COP course;
- MACD_t_mean – mean MACD_t value determined for the whole measurement;
- MACD_V – the value of the velocity of displacement changes between subsequent trend changes calculated as a ratio of displacement between subsequent points of the trend change (MACD_dS) to time MACD_t, calculated for the same points (dependence 1);
  MACD_V= MACD_dS / MACD_t(1)
- MACD_V_mean – mean MACD_V value determined for the whole measurement.

**Figure 2:**
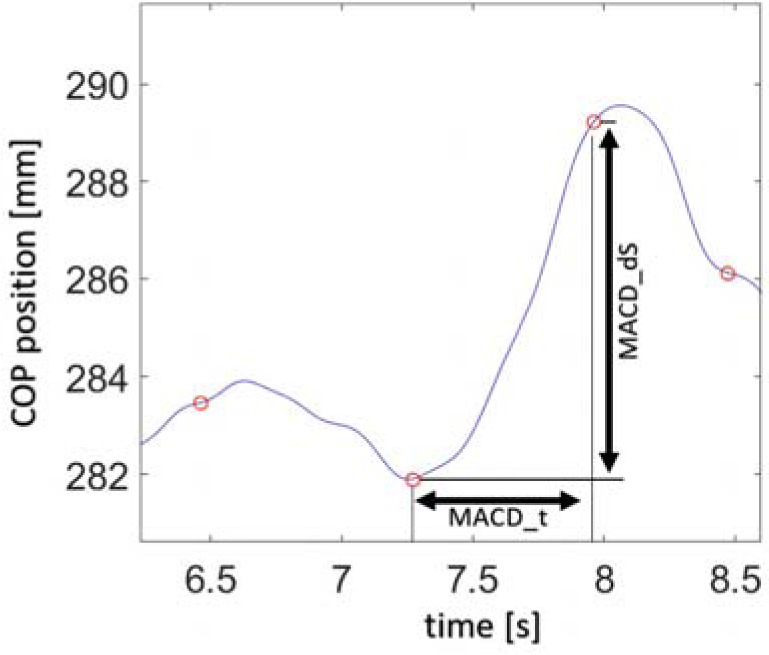
COP displacement signal with marked selected values calculated for two subsequent values of trend change points detected by the algorithm.

The physical values listed above can be determined for both the ML and AP directions. Visual interpretation of selected values is represented in Figure 2.

The methodology proposed by Wodarski et al. (2022) makes it possible to apply the trend change analysis of COP signals to the creation of trend change histograms, which are directly related to frequency analyses. It is possible to group detected trend changes based on MACD_t. Histograms created in this way show the number of detected trend changes between which the MACD_t time was contained in a defined time period. In the current study, it was decided to group MACD_t into time periods lasting between 0.0 s and 1.0 s with a step of 0.1 s. The frequency of occurrence of fast and slow changes could then be determined. During the analysis of the measurements, it was decided that MACD_t values lower than 0.1 s, that is with a frequency higher than 5 Hz, would be treated as non-significant in the COP signal (Marco-Ahulló et al., 2022). An upper boundary of 1.0 s resulted from the fact that in recorded changes of the COP location, the computation-applied parameters of the MACD determination did not create the possibility to determine single trend changes for which the time would be longer than 1.0 s. Therefore using dependence 2 for the proposed time periods, it was possible to calculate their respective frequency periods.

f = 1 / (2tp) (2)

where: f = frequency, tp = time between consecutive detected trend changes, with 2tp = period

**Figure 3:**
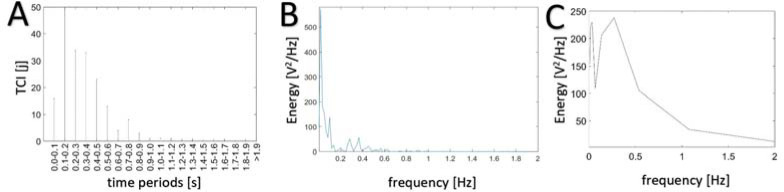
The number of trend changes in a time unit – TCI histogram (A), PSD signal (B) and signal energy in wavelet bands (C) illustrated by an example of COP signal in the AP direction in the case of free standing with open eyes. The results from the vertical axis may be calculated in the form of time periods and frequency using dependence 2.

Figure 3 shows an example of the analysis of a TCI histogram plotted for the course of the COP signal changes measured during a 60-s standing test and compares this with the frequency analysis.

Analysis of the data presented in Figure 3 provides a visual comparison of the TCI histogram distribution to the frequency analyses. In the COP signal measured, one can observe the concentration of the PSD signal and energy calculated based on wavelet analyses is near low frequencies. The lower the frequency, the higher the energy value. Similar frequency diagrams were obtained in previous research (Jurkojć, 2018; Michnik et al., 2014; Morasso, 2020; Paillard et al., 2006; Quijoux et al., 2021; Wodarski et al., 2021). Due to non-ideal filters of wavelet decomposition, lower energy values were distributed in a broader frequency band in the wavelet analysis (Figure 3C) than those that were observed in the PSD analysis (Figure 3B). The results show that during the activity of free standing, the number of cyclic (sinusoidal) components for the signal diminished with frequency, however, it was not possible to obtain unambiguous information on how these components were mapped from the shape of the tested COP signal in the time domain. In the analysis of the TCI histogram, the results should be interpreted as the number of trend changes in the COP displacement signal, which are distant from each other by a certain time interval read out on the horizontal axis of the diagram in the presented histogram. The results obtained for an example signal show a large number of fast trend changes and a gradually decreasing number of trend changes along with increasing time interval values.

COP displacement courses were obtained in both the AP and ML axes, and the following parameters were then determined for each tested participant: path, COP mean velocity (V), area, range of motion, TCI index, the MACD_t¬_mean, and MACD_V_mean.

For all analyzed data in the whole study group, the following values were calculated: mean (Mean), standard deviation (SD), coefficient of variation (CV) in percentage, median (M), half of inter-quartile range (QR/2), and quartile-based coefficient of variation as a ratio of QR/2 to Mean expressed as a percentage.

The next stage involved the calculation of the TCI histograms in an interval up to 1 s for the results obtained for each tested participant. The values were averaged and compared in respective periods between the OE and CE tests.

### Statistical Analysis

All analyses were performed using Matlab R2022a (MathWorks, MA, USA) and Statistica 13 (TIBCO, CA, USA) software. The Kolmogorov-Smirnov test was used to determine data normality of the parameters analysed. Normal distribution was observed only for the TCI. For the TCI, there was also homogeneity of variance tested with the Brown-Forsythe test. Differences between the means were investigated using the independent samples t-test. For the non-normally distributed quantities, the U Mann-Whitney test was used. High reliability of the results was obtained for all comparisons

## Results

Results from the conducted computations are presented in Table 1. Values obtained for the OE and CE tests were compared.

**Table 1:** Values of the variables analyzed during standing tests with open and closed eyes. Statistically significant results are marked with an asterisk (*) in the last column.

Quantities	Unit	OE						CE						*p*
		Mean	SD	CV [%]	M	QR/2	QCV [%]	Mean	SD	CV [%]	M	QR/2	QCV [%]	
Path	[mm]	172.8	53.6	31.0	163.3	31.5	19.3	214.4	66.3	30.9	211.9	45.0	21.2	0.01 (*)
Path AP	[mm]	124.3	39.8	32.0	116.5	26.9	23.1	166.4	54.8	33.0	160.8	30.4	18.9	0.01 (*)
Path ML	[mm]	97.8	34.8	35.6	94.4	22.1	23.4	103.9	36.1	34.8	99.7	25.1	25.1	0.88
V	[mm/s]	5.76	1.8	31.0	5.44	1.1	19.3	7.15	2.2	30.9	7.06	1.5	21.2	0.01 (*)
V AP	[mm/s]	4.14	1.3	32.0	3.88	0.9	23.1	5.55	1.8	33.0	5.36	1.0	18.9	0.01 (*)
V ML	[mm/s]	3.37	1.2	35.6	3.04	0.7	23.4	3.46	1.2	34.8	3.32	0.8	25.1	0.88
Area	[mm^2^]	158.9	118.5	74.6	121.3	63.7	52.5	132.8	91.6	69.0	106.1	45.7	43.0	0.14
Range of Motion AP	[mm]	18.03	7.0	38.7	16.51	3.6	22.1	18.67	6.1	32.6	17.32	4.0	22.9	0.33
Range of Motion ML	[mm]	10.71	6.1	56.6	9.46	3.1	33.2	9.83	4.6	46.6	8.88	3.3	37.6	0.78
TCI_AP	[no]	91.8	9.6	10.5	91.5	6.9	7.5	87.6	8.2	9.3	86.0	4.6	5.3	0.00 (*)
TCI_ML	[no]	108.7	14.1	13.0	109.0	9.4	8.6	108.5	15.3	14.1	107.0	11.6	10.9	0.98
MACD_t_mean_AP	[s]	0.33	0.03	10.4	0.32	0.02	7.3	0.31	0.03	9.0	0.31	0.02	5.9	0.01 (*)
MACD_t_mean_ML	[s]	0.28	0.04	13.7	0.27	0.0	8.3	0.28	0.04	14.0	0.28	0.03	10.5	0.99
MACD_V_mean_AP	[mm/s]	3.47	1.1	33.0	3.18	0.7	23.3	4.75	1.6	33.1	4.71	0.8	17.9	0.00 (*)
MACD_V_mean_ML	[mm/s]	2.45	1.0	40.8	2.22	0.6	25.3	2.70	1.1	39.0	2.56	0.8	29.9	0.38

**Figure 4:**
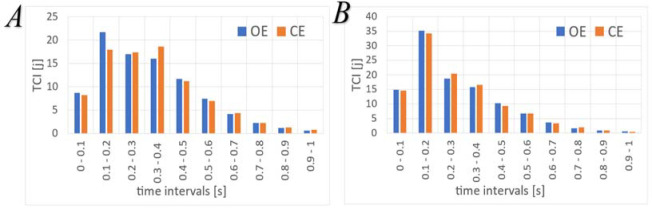
Mean TCI histograms in the OE and CE tests. Diagram A for the AP direction, diagram B for the ML direction.

Mean TCI histograms calculated for the OE and CE tests in the AP and ML directions are presented in Figure. 4. The diagrams were limited to an interval of 1 s due to the values close to 0 for time values greater than 1 s.

Values obtained from the OE and CE tests were compared for each time period. Statistically significant differences were only obtained for the AP axis, and the results are presented in Table 2.

**Table 2:** Time intervals in which statistically significant differences were obtained for the AP direction. Statistically significant results are marked with an asterisk (*).

time intervals [s]	0–0.1	0.1–0.2	0.2–0.3	0.3–0.4	0.4–0.5	0.5–0.6	0.6–0.7	0.7–0.8	0.8–0.9	0.9–1
*p*	0.06	0.02 (*)	0.75	0.03 (*)	0.38	0.24	0.42	0.74	0.57	0.32

## Discussion

### Analysis in the Time Domain

Analysis of stabilographic values, including the path, V, area, and range of motion, is standard in the assessment of functionality and the ability of the human body to maintain balance (Puszczałowska-Lizis et al., 2016; Wilczyński et al., 2018). Until recently, the above-mentioned values were the main variables analyzed in determining the individual’s condition, their level of dysfunction, and the progress of postural stability rehabilitation. From the results presented in Table 1, for a uniform study group of 118 participants, only the path, path in the AP direction, V, and V in the AP direction, showed significantly different values for the OE and CE tests. These mean values increased, which may be an indication of a change in strategy for maintaining balance during the CE test. This increase was recorded for participants of all ages in the CE test, which is in line with studies by Bibrowicz et al. (2018), Mańko et al. (2016) and Błaszczyk (2008). This increase was caused by switching off one of the mechanisms of feedback required for maintaining balance, which in this instance was eyesight (Takakusaki, 2017; Wodarski et al., 2022).

A slightly different situation occurred for measurements of range of motion and the area. Similarly to the results obtained by Błaszczyk (2008), the range of motion in the AP and ML directions did not reveal significant differences, which indicates that those values were not subject to change. Also the mean values for the area did not show significant differences between the OE and CE measurements. However, CV and QCV values expressed in percentages were approximately twice as high as in the path and V measurements. Nonetheless, analysis of the area values did not provide reliable results due to the excessive scatter of the data.

Statistical analysis of the investigated variables (Table 1) revealed no significant differences in the ML direction between the OE and CE conditions. Similar results were obtained by Wilczyński et al. (2018) for the young study participants, and by Błaszczyk (2008) who investigated an elderly population, where the range of motion, V, path and area did not show any increase in CE tests compared to OE tests in the ML direction.

Computations performed in line with TCI analysis methodology for the MACD_t, MACD_V, and TCI variables revealed significant differences only in the AP direction. However, attention should be paid to the fact that CV and QCV values for TCI AP and MACD_t_mean in the AP direction were three times lower than all of the other analyzed values. These values ranged from 9% to 14% for CV, and from 5.9% to 10.9% for QCV The largest concentration of mean and median values represent the greatest sensitivity of these values if irregularities were to occur in the assessment of human balance disorders. Meanwhile, low CV and QCV values indicate that these indexes are sensitive and can detect slight deviations from a set of standards, meaning these variables are suitable indexes for the evaluation of postural stability.

An increase of over 20% was recorded for the mean values of the path (from 124.3 mm to 166.4 mm) and V (from 4.14 mm/s to 5.55 mm/s) in the AP direction. However, no increase was observed for range of motion in the AP direction. These increases indicated a trend change in COP displacement values between subsequent postural corrections. This observation was confirmed by a small number of posture corrections and the time between those corrections, where the TCI and MACD_t values changed by less than 5% (from 99.8 to 87.6) and 6% (from 0.33 to 0.31), respectively. The prolongation of displacement between subsequent postural corrections was also confirmed by the increased velocity of MACD_V_mean in the AP axis from 3.47 mm/s to 4.75 mm/s, and a slight decrease in MACD_t from 0.33 s to 0.31 s.

Range of motion in the AP axis did not increase in the CE tests in comparison to the OE tests. However, the amplitudes and velocity of smaller oscillations from the equilibrium level changed, indicating a different strategy for maintaining balance. The strategy for the CE condition was revealed by a smaller number of quick corrections and an increase in the corrections, which lasted for a longer period of time (Figure 4A). Moreover, there was a prolongation of the displacement between subsequent posture corrections in relation to the OE condition, and most importantly, there was an increase in the mean velocity values between subsequent changes in the trend. This may be of great importance for assessing the risk of falling.

To confirm the change in the balance-keeping strategy in the AP axis, it was necessary to conduct an analysis of the TCI histograms.

### Analysis of the TCI Histograms

Analysis of histograms provided information on the number of trend changes that occurred in subsequent time intervals between two neighboring trend changes. These periods were determined with a step of 0.1 s and were limited to the value of 1 s. These variables were adopted to detect subsequent trend changes and to exclude two subsequent trend changes of longer than 1 s. The results were analyzed and compared to the TCI values in the respective time periods for the OE and CE conditions. The ML axis did not show any significant differences.

The analysis of the TCI histogram for the AP direction (Figure 4A) indicated that the time interval of 0.1–0.2 s resulted in a higher number of significant postural corrections in the OE tests compared to the CE tests. For the time period between 0.2 and 0.3 s, the TCI showed equal values for both measurement conditions, whereas in the period between 0.3 and 0.4 s, the CE test had more corrections compared to the OE test. During time intervals greater than 0.4 s, and between 0 and 0.1 s, the results did not reveal any significant differences between mean values. Therefore, the balance-keeping strategy connected with eye closure resulted in an increased number of slower posture corrections between 0.3 and 0.4 s, and a decrease in corrections between 0.1 and 0.2 s (Table 2). Thus, it was concluded that a smaller number of quick postural corrections, connected with changes in the balance-keeping strategy, indicated by an increase in slight oscillations in the COP course, did not influence the range of motion obtained during the whole examination. Changes in TCI histograms at low time values were also observed in previous research using PSD analysis (Wodarski et al., 2022). Similarly, in research by Jurkojć (2018), it was observed that the share of harmonics of higher frequency increased after eye closure, and there was increased amplitude in the bandwidth over 0.4 Hz. The same phenomenon was also observed in Wodarski et al. (2020), where wavelet analysis was used to determine the energy in individual frequency bands of the COP signal. In the present study, an increase in energy in the bands of higher frequency, which is connected with an increase in the amplitude of the signal in these bands, was demonstrated. Such growth of important components that occurred in the medium period of the analyzed frequencies (which corresponds to medium time values in the TCI histogram of 0.4–0.6 s) after eye closure was also noted in a study by Iuppariello et al. (2016), in dynamic test conditions by Liang et al. (2014) and in patients with neurological problems investigated by Cabez-Ruiz et al. (2011).

The advantage of TCI over PSD and wavelet analysis is the high resolution of the values obtained, which results from the freedom of choice over the boundaries of time intervals. It should also be emphasized that analyses may be grouped into time periods of different breadth, which is impossible with wavelet analysis, where periods are strongly dependent on the sampling frequency of the signal. As for TCI analysis, it can detect trend changes that may not only be noncyclical, but are also independent of the sinusoidal constituents of the signal and thus, not connected with the shape and choice of wavelet, as is the case for PSD and wavelet analyses. Moreover, this type of analysis is performed in the domain of time, which makes it insensitive, for instance, to the phenomena of spectral leakage.

Considering all the advantages of the proposed calculation method, its limitations should also be taken into account. The applied calculation algorithm and the applied coefficients enable a thorough analysis of high-frequency trend changes, which, however, results in limited possibilities of analyzing low-frequency trend changes. The proposed method of calculations also requires taking into account the influence of other methods of selection of trend change points on the COP signal and comparing the influence of the choice of selection method on the obtained accuracy of trend change indications.

## Conclusions

The proposed methodology of trend changes analysis, based on the determination of points of trend change in COP displacements, makes it possible to supplement traditional methods of analysis with additional information connected to strategies used by humans to maintain balance. Using the trend change analysis method, differences in the strategy for maintaining balance between the OE and CE tests were observed. Indeed, results from the time domain provided information on the frequency of occurrence of postural corrections as well as COP movement velocity between such corrections. The conclusions drawn support information connected with changes in the path, V, and area, in addition to information on the number of posture corrections, the time between such corrections, and the velocity of COP movement between such corrections.

The algorithm used to determine points of trend change, the TCI, MACD_t, and MACD_V indexes, as well as the TCI histograms, are based on analysis in the time domain. Thus, the results were not distorted by noise, as is seen with spectral leakage in FFT analysis. These indexes can be determined in real-time and provide information on trend changes in the signal, which may be useful in the investigation of fall prediction. Furthermore, sections of analysis in the histogram may be freely selected, which is a considerable advantage in comparison with wavelet analysis, where they depend on the sampling frequency or parameters of a given base wavelet. The analysis of TCI histograms confirms the results obtained from PSD, FFT, and wavelet analyses, in connection with changes to the balance-keeping strategy after eye closure.

Slight SDs and small interquartile ranges (from 5% to 14%) for the TCI and MACD_t measurements in both the OE and CE tests classify these variables to be indexes of posture stability that are highly sensitive to small changes. A slight deviation of values, such as those connected with neurological issues, that impact changes to postural stability, may be discovered more quickly using these methods compared to the analysis of the path or area variables.

The proposed computational method based on the analysis of trend changes constitutes a tool that can find clinical application. Furthermore, it does not require a verification step to ensure that the number of trend changes in the COP signal correlates with the level of stress or the level of nervous system stimulation.
